# Effect of amniotic over collagen membrane and buccal fat pad in treating oral sub-mucous fibrosis

**DOI:** 10.6026/9732063002001760

**Published:** 2024-12-31

**Authors:** Asha S Badadesai, Nureldeen AN Elhammali, Pradeep Kumar Sharma, Madhvika Patidar, Kannu Sharma, Nasim, Sohini Deb, Vardarajula Venkat Ramaiah

**Affiliations:** 1 Department of Oral and Maxillofacial Surgery, PMNM Dental College and Hospital, Bagalkot - 587103, Karnataka, India; 2 Department of Oral and Maxillofacial Surgery, Sirte University, Libya, North Africa; 3 Department of Oral and Maxillofacial Surgery, Government Dental College, Dibrugarh, Assam, India; 4 Department of Oral Pathology and Microbiology, Government College of Dentistry, Indore, Madhya Pradesh, India; 5 Department of Oral & Maxillofacial Surgery, Inderprastha Dental College and Hospital, Ghaziabad, India; 6 Intern, Kalinga Institute of Dental Sciences, Bhubaneswar, Odisha, India; 7 Department of Dental Hygiene, College of Applied Health Sciences, Ar Rass, Qassim University, Saudi Arabia

**Keywords:** Buccal fat pad, collagen membrane, amniotic membrane, oral sub-mucous fibrosis

## Abstract

Oral submucous fibrosis (OSMF) is a chronic condition that involves any portion of the oral cavity and causes restricted mouth opening due to increasing sub mucosal fibrosis. Various surgical treatment approaches have been used to correct the OSMF. Therefore, it is of interest to compare the efficiency of amniotic membrane, collagen membrane and buccal fat pad in OSMF patients. Hence, a total 30 patient's diagnosed with OSMF were alienated equally into 3 groups based on treatment method; Group I: buccal pad of fat (BPF), Group II: mucosal defect with amniotic membrane (AM) and Group III-xenogenous collagen membrane (CM). Results show that AM grafts are advisable alternative for oral tissue restoration than buccal fat pad flaps and collagen membranes in terms of discomfort reduction, mouth opening improvement and epithelisation.

## Background:

Oral submucous fibrosis (OSMF) is a condition with an unknown aetiology that is commonly observed in India [1]. OSMF is classified as a precancerous, potentially malignant disease which can develop into cheek carcinoma. Any area of the oral cavity, including the throat, may be involved by this harmful, long-term condition [1, 2]. It is always linked to a juxtaepithelial inflammatory reaction, followed by a fibroelastic alteration in the lamina propria with epithelial atrophy, which causes oral mucosal stiffness, trismus and inability to eat, even though it is sometimes preceded by and/or associated with vesicle formation [3]. Capsaicin, hypersensitivity, areca nut alkaloids, autoimmune, hereditary vulnerability and chronic zinc, iron and vitamin B complex insufficiency are some of the etiological factors that have been suggested by different authors [2, 4]. However chewing irritants like tobacco, areca nutand (Areca catechu) betel leaf frequently causes it [5]. These substances promote the synthesis of collagen, a hard, fibrous protein that fortifies the mouth cavity's muscles and sensitive mucous membrane [5]. Clinically, OSMF is defined by a burning feeling in the mouth after eating spicy foods, vesicles, recurring stomatitis, impaired gustatory sensitivity and initial mouth dryness. Pain on probing in the areas where sub mucosal fibrotic bands are developing is an important clinical symptom and trismus is mostly caused by fibrosis in the thick tissue around the pterygomandibular raphe [2]. Scarring is seen, along with mucous membrane atrophy and swallowing pain. Atrophic mucous may ulcerate often, leading to cancer [7]. Late signs include difficulties in opening the mouth. Histo-pathologically, it is distinguished by increasing sub mucosal fibrosis and epithelial alterations that range from atrophy to hyperplasia to dysplasia [2].

The World Health Organisation defines a precancerous oral disease as "a generalised pathological state of the oral mucosa associated with a significantly increased risk of cancer", which closely matches the criteria of OSMF [8]. The probability of malignant transformation in OSMF patients is between 3 and 6% [6]. Various therapies for OSMF have been offered in an attempt to enhance mouth opening such as medications (conservative measures) and surgery, with different degrees of success [9]. The primary goal of any therapy strategy for OSMF cases is to alleviate symptoms such as burning in the mouth, ulceration and rigidity of the oral mucosa and to improve mouth opening [6]. Relapse is a common complication of surgically releasing the oral trismus produced by OSMF. Fibrous bands are often removed during surgical care of OSMF, followed by the placement of appropriate grafts such as a buccal pad of fat (BPF), collagen membrane (CM), or more recently with amniotic membrane (AM)[2, 6, 9].Various flaps are employed to close OSMF defect. Bilateral palatal flaps result in a huge bare region on the palatal bones. When a significant defect is developed, local flaps may be insufficient to cover the completed efficiency. A nasolabial flap is insufficient to conceal the defect and leaves obvious scars on the face. Tongue flaps were employed to hide the buccal deformities; however these proved to be cumbersome. Hence alternative grafts were tried. Buccal fat pad is distinguished by its anatomical position and ease of access and mobilisation without generating any visible defect [6]. The use of BFP for treatment of OSMF has proven to be highly effective. Collagen is a biological product with the advantages of being less antigenic, having high tissue compatibility and being readily available [2]. The amniotic membrane (AM) is a colourless, transparent membrane that is free of blood vessels, nerves and lymphatics. It is derived from the placenta's deepest layer. AM are a versatile and useful choice in maxillofacial surgery, as they promote wound healing, reduce inflammation and serve as a scaffold for tissue regeneration [10]. Amnion allografts are membranes that envelop and protect embryos by forming an amniotic sac. Amnion allograft has been employed in medicine due of its excellent wound modifying capabilities. In the realm of dentistry, only a few papers have presented its properties in the healing of oral wounds [11]. Fresh amnion has been widely employed, although dried, frozen, irradiated and lyophilised preparations have also been tested. The mesenchymal surface of the AM will be placed to the wound site [12]. The AM can be used to close post-surgery defects in patients with oral submucous fibrosis, allowing it to use its growth factor and scaffolding properties to promote healing. Therefore, it is of interest to compare the effectiveness of collagen membrane, BFP and freeze-dried amniotic membrane as grafting materials in the surgical treatment of oral submucous fibrosis.

## Materials and Methods:

After receiving ethical approval from the relevant authorities and participant informed agreement, the current investigation was conducted in the Oral and maxillofacial Surgery department. The study comprised patients with proven OSMF who were reported to the Oral and maxillofacial Surgery outpatient department. Total 30 patents with OSMF were arbitrarily alienated into 3 groups with 10 samples in each group based on treatment method; Group I:buccal pad of fat (BPF), Group II: mucosal defect with amniotic membrane (AM) and Group III-xenogenous collagen membrane (CM) 5x5 size (EUCARE Pharmaceuticals Private Limited). Healthy individuals aged 20-55 years with mouth opening less than 25 mm, having grade II and Grade III OSMF cases were considered for this research. Each patient provided a thorough history, paying particular attention to their duration and behaviours.

## Surgical procedure:

The preoperative and postoperative mouth openings (inter incisor distances) in both groups were measured in millimetres. The fibrous bands were palpated to determine the size of the incision. The incisions were performed bilaterally on each side of the buccal mucosa with a no. 15 Bard Parker blade. The incision reached posteriorly to the pterygomandibular raphe (or anterior pillar of the fauces) and anteriorly to the angle of the mouth. The incision was made to the depth of the submucosal layer. In group I, fibrotomy of the bands was performed using blade no. 15. A bilateral coronoidectomy was carried out, addressing the coronoid processes with the same incision. The interincisal opening was measured after the bilateral extraction of the maxillary and mandibular third molars. Depending on the magnitude of the defect, the BPF was typically gathered at a length of 3 cm and a breadth of 4 cm. The flap was sutured after the exposed portion was covered with BFP. Following the fibrotomy, Group II employed a freeze-dried irradiated amniotic membrane to cover the complete defect. Similarly, in Group III, after the surgical procedure, the xenogenous collagen membrane material was reconstituted by immersing it in normal saline for 5 minutes before being trimmed to the desired form using scissors and suturing the region. Physiotherapy began on the fifth postoperative day and continued for six months. Patients were recalled until epithelialisation was complete and then for frequent follow-ups at 1, 3 and 6 months. The method's performance was evaluated using preoperative and postoperative mouth opening, rate of epithelisation and pain assessment using a VAS (visual analogue scale) scale ranging from no pain to mild, moderate and severe pain with a score of 0 to 10. ANOVA and the Chi square test at P<0.05 were used in the statistical analysis of the collected data using SPSS software version 22.0.

## Results:

Measuring the mean mouth openness before surgery, during surgery and at one, three and six months showed a considerable improvement. The mouth opening was better with amniotic membrane group followed by collagen membrane and BPF (Table 1). Epithelisation was faster in days among AM followed by CM and BPF (Table 2). The mean post-operative pain score did not appreciably vary between the groups; however, the AM and collegiate membrane groups experienced lower pain scores than the BPF group (Table 3).

## Discussion:

OSMF is a chronic, progressive precancerous disease of the oral mucosa. The Indian subcontinent is the primary location for OSMF [6]. Many conservative therapy options for OSMF have been offered, including oral treatment with antioxidants, vitamins, iron supplements and zinc. Iodides and different intralesional injections were utilised, including hyaluronidase, collagenase and hydrocortisone [9]. Surgery is the preferred approach for people who have a significant limitation in mouth opening. Following surgical procedures to cover bare areas, temporalis myotomy, tongue flaps, palatal island flaps, bilateral nasolabial flaps, split-thickness skin grafting and coronoidectomy were suggested. When trismus is severe, surgery can be helpful [9]. In the present research, we assessed the clinical efficiency of BFP with collagen and AM in the restoration of postoperative defects in OSMF. We discovered that all treatments are successful in lowering postoperative discomfort, improving defect epithelisation and improving the mouth opening, although the amnion membrane was the most beneficial, followed by the college membrane and the buccal pad of fat. In this study, all third molars were extracted prophylactically to avoid stress to the retromolar flap. The trend of pain reduction with time is indicative of adequate healing in the research groups and it is consistent with the findings of Yeh *et al.* In Yeh's study, the mean mouth opening was increased postoperatively [13]. It was discovered that harvesting the BFP did not result in any noticeable deformity in the cheek. In terms of the oral cavity, buccal fat pad harvesting is a simple technique. The BFP is not entirely free of complications. It can induce significant atrophy [9]. According to Shaikh *et al.* buccal fat pads work well as pedicled grafts in the surgical treatment of oral submucous fibrosis. The post-surgical recovery is satisfactory [14]. Randhawa *et al.* investigated the efficacy of BFP and collagen membrane as inter-positional materials in OSMF surgical therapy. They concluded that the collagen membrane was more efficient than BFP [6]. The results are consistent with our findings. In contrast to our findings, Pandey *et al.* determined that BFP demonstrated faster epithelisation and less wound contracture than collagen membrane [2]. Movaniya *et al.* proposed college membrane as a viable option to existing graft materials for the healing of mucous membrane lesions [15]. Rastogi *et al.* conducted a prospective research of sixty subjects in which he used collagen membrane in surgical defects and discovered it to be a superior option compared to other graft materials for repair [16]. According to Poddar *et al.* platelet rich fibrin (PRF) is a better dressing material than Collagen Membrane and has a higher healing capability for treating various oral mucosal lesions [17]. In treating OSMF, Pradhan *et al.* determined that collagen membrane is a preferable approach compared to transplantation of the BPF as a graft to cover the surgical lesion [18]. Sharma *et al.* evaluated the efficacy of AM with BPF for OSMF and reported that AM was easy to handle and with adequate pain management [9]. Boricha *et al.* compared AM with respect to pain relief, wound healing and membrane safety, concluding that pain alleviation and healing were much better in AM instances and membrane safety was equally good [19]. Choi and Tseng discovered that the amniotic membrane reduces the expression of TGF-b receptors in fibroblasts, resulting in reduced fibrosis. There was no significant difference in the distribution of swelling status on the left and right sides utilising BFP and amniotic membrane, respectively [20]. Kothari *et al.* determined that amniotic membrane grafts are feasible and reliable for covering the defect surface [21].

Wounds left uncovered are susceptible to infection and scarring. For nearly a century, general surgeons have known that grafted wounds heal faster and with fewer problems than open wounds. Mucosal grafts may provide the solution since they come closest to meeting the requirements of an ideal graft material [15]. In our investigation, no instances had adverse or allergic reactions to the BPF, amnion membrane, or collagen, demonstrating its safety as a grafting material. The majority of BFP in our study was determined to be adequate in all cases and it retained its position. Collagen dressing employed in the study is a biological dressing made of type I and type III bovine collagen, which is identical to human collagen. The collagen membrane demonstrated good adaptation to the surgical defect. Clinically, collagen is well tolerated and has no side effects [15]. Further researches are necessary to confirm the findings on larger sample size.

## Conclusion:

The amniotic membrane resilience encourages the prompt initiation of mouth opening activities, leading to better mouth opening than with the BFP flap. For the BFP, spontaneous epithelisation provides a more sensible, reliable and useful surgical method. The amniotic membrane showed good haemostatic qualities and was simpler to use than BPF.

## Figures and Tables

**Table 1:** Comparison of mean mouth opening

**Groups**	**Pre operative**	**Intra operative**	**1 month**	**3 month**	**6 month**	p
Group I - BPF	14.2	38.4	24.2	30.3	31.6	
Group II - AM	14.6	38.6	27.5	34.2	34.6	
Group III - CM	14.7	38.5	26.4	33.4	33.8	0.001

**Table 2:** Epithelisation rate in days

**Groups**	**Epithelisation**	**Days**
Group I - BPF	26.40%	28
Group II - AM	13.50%	14
Group III - CM	16.20%	16

**Table 3:** Mean post-operative pain score

**Groups**	**Pain present**	**Pain absent**
Group I - BPF	18.60%	81.40%
Group II - AM	6.20%	93.80%
Group III - CM	8.40%	91.60%
